# The significance of spleen size in children with sickle cell anemia

**DOI:** 10.1002/ajh.26703

**Published:** 2022-09-27

**Authors:** Amina Nardo‐Marino, Andreas Glenthøj, John N. Brewin, Jesper Petersen, Thomas H. Braunstein, Jørgen A. L. Kurtzhals, Thomas N. Williams, David C. Rees

**Affiliations:** ^1^ Danish Centre for Haemoglobinopathies, Department of Haematology Copenhagen University Hospital, Rigshospitalet Copenhagen Denmark; ^2^ Department of Immunology and Microbiology, Centre for Medical Parasitology University of Copenhagen Copenhagen Denmark; ^3^ Department of Haematological Medicine King's College Hospital London United Kingdom; ^4^ Comprehensive Cancer Centre, School of Cancer and Pharmaceutical Sciences King's College London London United Kingdom; ^5^ Core Facility for Integrated Microscopy, Faculty of Health and Medical Sciences University of Copenhagen Copenhagen Denmark; ^6^ Department of Clinical Microbiology Copenhagen University Hospital, Rigshospitalet Copenhagen Denmark; ^7^ KEMRI‐Wellcome Trust Research Programme Kilifi Kenya; ^8^ Department of Surgery and Cancer Institute of Global Health Innovation, Imperial College London London United Kingdom

## Abstract

It is well established that splenic dysfunction occurs in early childhood in sickle cell anemia (SCA), although the determinants and consequences of splenic injury are not fully understood. In this study, we examined spleen size and splenic function in 100 children with SCA aged 0–16 years at King's College Hospital in London. Spleen size was assessed by abdominal ultrasound (US) and splenic function by pitted red blood cells (PIT counts). In our cohort, 5.6% of children aged 6–10 years and 19.4% of children aged 11–16 years had no visible spleen on US (autosplenectomy). Splenomegaly was common in all age groups, with 28% of children overall having larger spleens than the average for their age. Only one child had a PIT count suggesting preserved splenic function. We found no correlation between hemoglobin F levels and spleen size, nor was there any difference in spleen size between children treated with or without hydroxyurea. Although there was a trend toward increased spleen length in children with co‐inherited α‐thalassemia, this did not reach statistical significance. Finally, we found a strong association between erythrocyte deformability measured with oxygen gradient ektacytometry, spleen size, and PIT counts. In conclusion, our results do not agree with the general perception that most children with SCA undergo autosplenectomy within the first decade of life and indicate that loss of erythrocyte deformability contributes to loss of splenic filtration capacity in SCA, as well as phenotypical variations in spleen size.

## 1 BACKGROUND

The spleen is one of the first organs affected in sickle cell anemia (SCA).^1^ Sickled cells cause splenic ischemia in early childhood, ultimately leading to irreversible damage of the spleen. It is well‐established that splenic function is lost within the first years of life ^2^, ^3^, ^4^ and that hyposplenia is a significant feature of SCA. Lack of splenic function is associated with severe and potentially fatal complications, including overwhelming infections with encapsulated bacteria.^5^, ^6^ Another life‐threatening complication in SCA is acute splenic sequestration (ASS), a rapid pooling of blood in the spleen causing sudden splenic enlargement with progressive anemia and hypovolemia.^7^

Spleen size varies significantly in SCA, the determinants and consequences of which are not fully understood. Transient splenomegaly is often described in early life, typically followed by progressive splenic fibrosis and atrophy (autosplenectomy).^8^, ^9^ It is widely believed that autosplenectomy is mostly complete within the first decade of life, particularly in children living in Europe or North America.^10^ In contrast, splenomegaly during later life has more often been described in sub‐Saharan Africa (SSA). While this finding has frequently been attributed to malaria,^11^, ^12^ multiple genetic and external factors may contribute. High levels of hemoglobin F (HbF) and co‐inheritance of α‐thalassemia have also been associated with splenomegaly,^13^, ^14^ as has the presence of irreversibly sickled cells (ISCs).^8^ Other infectious agents known to cause splenomegaly may further interfere with spleen size.

In the current study, we examined spleen size and splenic function in a cohort of children with SCA at King's College Hospital in London. We assessed factors that could potentially influence spleen size, including HbF, α‐thalassemia genotype, hydroxyurea (HU) therapy, red blood cell (RBC) deformability, and viral infections. We also investigated whether spleen size was associated with certain clinical complications.

## 2 METHODS

### 2.1 Study population

Children aged 0–16 years with a confirmed diagnosis of SCA were recruited from the pediatric hematology clinic at King's College Hospital in London between December 2018 and January 2019. Other aspects of this cohort have previously been studied.^15^ Children and their parents or guardians were approached when attending a routine clinic appointment. Children were selected at random during clinics, and only children with the HbSS genotype were included. Children who had previously undergone surgical splenectomy were excluded from the study. Other exclusion criteria were the receipt of HU therapy for less than 3 months, or if the dose had changed within this time, and the receipt of any blood transfusions within 3 months of recruitment.

### 2.2 Ethics

The study was approved by the National Health Service (NHS) Research Ethics Committee (ref: 18/LO/1566). Prior to study recruitment, a parent or guardian provided written informed consent for all children <16 years while, additionally, children aged 11–15 years provided written assent. All 16‐year‐old children provided written informed consent.

### 2.3 Data collection

#### 2.3.1 Clinical examination

A standard clinical examination was performed on all children as part of their routine clinic appointment. This included splenic palpation at the anterior axillary and mid‐clavicular lines to estimate spleen size. Palpable splenomegaly was reported in cm under the left costal margin in the mid‐clavicular line. All examinations were performed by a single examiner.

#### 2.3.2 Abdominal ultrasound

Spleen measurements were obtained by abdominal ultrasound (US) performed in the pediatric hematology clinic on the day of study recruitment. All scans were performed by a single examiner with a level 1 competence in gastroenterological US^16^ using Philips 795005 Lumify System (Philips Denmark A/S). In case of any abnormalities or questionable findings, scans were reviewed by a consultant radiologist with a level 3 competence in gastroenterological US.

US imaging included the maximum longitudinal (spleen length, *L*), anterior–posterior (*AP*), and transverse (spleen width, *T*) measurements of the spleen in the transverse and longitudinal planes. Spleen volume (ml) was calculated using the formula for an ellipsoid model: (*L* × *AP* × *T*) × 0.523.^17^, ^18^ Spleen length was prioritized in cases where children could not tolerate the full set of measurements. US images and measurements were all recorded in case they needed to be reviewed subsequently.

#### 2.3.3 Blood samples

Blood samples were collected from all children on the day of study recruitment. Samples were analyzed routinely at the King's College Hospital laboratory, including standard hematology parameters, Epstein–Barr Virus (EBV) viral capsid antigen (VCA)‐IgG, Cytomegalovirus (CMV) IgG, and parvovirus B19 IgG. HbF percentage (%HbF) was determined by high‐performance liquid chromatography (HPLC) using a Variant II (Bio‐Rad Laboratories). Glucose‐6‐phosphate dehydrogenase (G6PD) deficiency was diagnosed with a direct enzyme assay.^19^ Samples were screened for common α‐thalassemia deletions (3.7, 4.2, and 20.5 kb; SEA, MED, and FIL) using a multiplexed gap PCR assay.^20^

Samples for RBC deformability measurements were stored at 4°C and transported from King's College Hospital, London to Copenhagen University Hospital, Denmark, where they were analyzed using the Oxygenscan module of the Laser Optical Rotational Red Cell Analyzer (Lorrca, RR Mechatronics), as previously described.^15^

#### 2.3.4 Splenic function

Splenic function was assessed by manual pitted RBC counts as previously described.^21^ RBCs were fixed on the day of sampling by mixing 50 μl of EDTA blood with 2 ml 2% PBS‐buffered paraformaldehyde. Samples were transported from King's College Hospital, London to the Core Facility for Integrated Microscopy, Faculty of Health and Medical Sciences, University of Copenhagen, where microscopy was performed within 3 months of venepuncture.^2^, ^21^ For each sample, a minimum of 500 consecutive RBCs were counted while inspecting them for the presence of one or more pits (rounded crater‐like depressions on the cell surface). The proportion of RBCs with pits, often referred to as the PIT count, was expressed as a percentage of the total number of counted cells (%PIT). A %PIT of <3.5%, was interpreted as normal and a %PIT of ≥3.5% as showing reduced splenic function.^2^

#### 2.3.5 Medical records

After obtaining written informed consent, medical records were examined for all children. Information regarding current and past HU therapy was recorded, as were results of the most recently performed Transcranial Doppler (TCD) scan. Visits to the emergency department or hospital admissions, previous episodes of ASS, or acute chest syndrome (ACS) were also recorded. Any acute episode of pain leading to a hospital admission or emergency department visit was interpreted as an acute painful crisis. All children and their parents or guardians were asked about country of birth and travel history to malaria‐endemic countries.

### 2.4 Statistical methods

Continuous variables with normal distribution were reported as mean ± standard deviation (SD). For normally distributed data, linear correlations were calculated using Pearson's correlation and groups were compared using a two‐sided *t*‐test or one‐way ANOVA. For variables with non‐normal distribution, medians and interquartile ranges (IQRs) were reported and linear correlations were calculated using Spearman's correlation, while groups were compared using Mann–Whitney *U* test or Kruskal–Wallis *H* test. Continuous outcomes were examined using multiple linear regression and categorical outcomes were examined using the chi‐squared test. Significance level was defined as *p* < .05. All analyses were performed in Stata V16.1 (StataCorp).

## 3 RESULTS

### 3.1 Study population

We recruited 100 children with a confirmed diagnosis of SCA (HbSS) in the study. Ages ranged from 7 months to 16 years (mean age 8.4 years). Baseline data are presented in Table 1. In our clinic, only 1%–2% of children with SCA have undergone surgical splenectomy, and no children selected to take part in this study were excluded because of prior splenectomy.

**TABLE 1:** Baseline characteristics of study population (*n* = 100 unless otherwise indicated)

	Mean (range)
Age (years)	8.4 (7 months to 16 years)
Girls, *n* (%)	48 (48%)
HU treatment, *n* (%)	47 (47%)
**α‐thalassemia** **(3.7 kb deletion),** ***n*** **(%)**	
No α‐globin gene deletions	54 (54%)
Heterozygous (one α‐globin gene deletion)	36 (36%)
Homozygous (two α‐globin gene deletions)	7 (7%)
Not performed	3 (3%)
**G6PD**, ***n*** **(%)**	
Normal activity	84 (84%)
Deficiency	10 (10%)
Not performed	6 (6%)
**Laboratory values**	
HbF (%), *n* = 95	14.1 (0.4–36.4)
Hb (g/L), *n* = 97	87.3 (62–116)
Bilirubin (μmol/L), *n* = 97	37 (10–138)
LDH^a^ (IU/L), *n* = 79	569 (312–977)
Reticulocytes (%), *n* = 97	12.4 (2.7–36)
WBC (10^9^/L), *n* = 97	9.1 (3.7–15.8)
ANC (10^9^/L), *n* = 97	3.9 (1.1–9.9)
Platelets (10^9^/L), *n* = 97	355 (59–709)
Creatinine (μmol/L), *n* = 97	33.3 (18–74)
CRP (mg/L), *n* = 94	5.3 (0.6–72)
**Immunology**	
*Cytomegalovirus IgG*	
Positive	51 (51%)
Negative	44 (44%)
Not performed	5 (5%)
*Epstein–Barr virus IgG*	
Positive	57 (57%)
Negative	34 (34%)
Not performed	9 (9%)
*Parvovirus B19 IgG*	
Positive	15 (15%)
Negative	70 (70%)
Not performed	15 (15%)

Abbreviations: ANC, absolute neutrophil count; CRP, C‐reactive protein; G6PD, glucose‐6‐phosphate dehydrogenase; Hb, hemoglobin; HbF, hemoglobin F; HU, hydroxyurea; IgG, immunoglobulin G; LDH, lactate dehydrogenase; WBC, white blood cell count.

^a^ Normal value < 240 IU/L.

### 3.2 Spleen size

While spleen length was measured for all children, spleen AP and T measurements were additionally obtained for only 71. Spleen length ranged from 0 to 16.6 cm (mean ± SD: 8.5 ± 3.7 cm) and spleen volume from 0 to 736.6 ml (median: 65 ml, IQR: 99.8). There was a strong positive correlation between spleen length and spleen volume (*r* = .93, *p* ≤ .0001).

**FIGURE 1:**
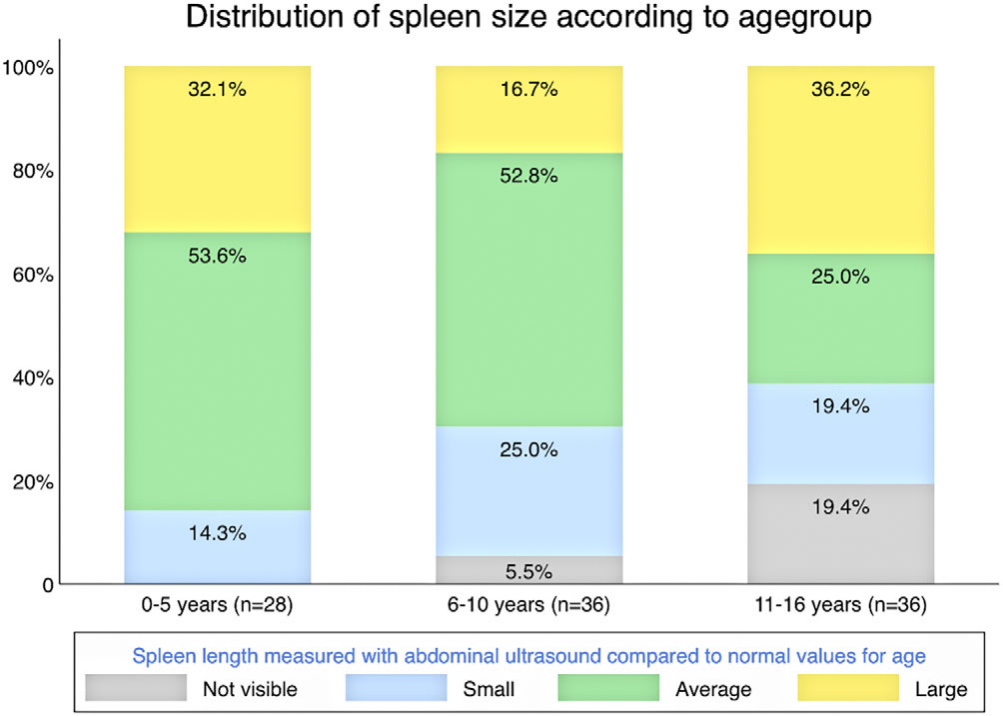
Distribution of spleen size in different age groups. Spleen size is assessed by spleen length measured with abdominal ultrasound. Results are compared to normal values for age.^22^ [Color figure can be viewed at wileyonlinelibrary.com]

Of the 100 children, 9 had no visible spleen by US, spleen length for age was <10th percentile in 20, average in 43 and above the suggested upper limit for age in 28.^22^ Data on spleen length compared to normal values for age are presented in Figure 1. The nine children who had no visible spleen were all ≥8 years of age, five were 15 years old. We found no significant correlation between spleen length and age (*r* = .10, *p* = .3) or spleen volume and age (*r* = .17, *p* = .2). Furthermore, there was no significant correlation between spleen length and height (*r* = .10, *p* = .3) or spleen volume and height (*r* = .21, *p* = .08).

On clinical examination, 18 children had palpable splenomegaly ranging from 1 to 8 cm below the left costal margin. Of these, 14 had a spleen length above the suggested upper limit for age when measured with US. For the remaining four children, the spleen was only palpable 1 cm under the costal margin and was of average length for age. Notably, spleens were not palpable on clinical examination in 14 of the 28 children with spleen lengths above the upper limit for their age. Nonetheless, when examining the association between palpable splenomegaly on clinical examination (yes/no) and spleen length for age measured with US (not visible/small/average/large), the relationship was significant (*p* < .001).

There was a weak negative correlation between spleen length and platelet count (*r* = −.29, *p* = .004). There was no significant correlation, however, between spleen length and hemoglobin or absolute neutrophil count.

### 3.3 Splenic function

Splenic function was assessed in 99 children. %PIT ranged from 2% to 57.3%, with a mean of 33.8%. Only one child (aged 1 year) had a %PIT of <3.5%, suggestive of normal splenic function. We found a significant positive correlation between %PIT and age (*r* = .41, *p* < .001) and a significant negative correlation between %PIT and %HbF (*r* = −.42, *p* < .001). When calculating a multiple linear regression model with age and %HbF as co‐factors, both were found to be significant predictors of %PIT (age: *p* = .002, %HbF: *p* = .007). Neither spleen length nor spleen volume was a significant predictor of %PIT.

### 3.4 Determinants of spleen size

#### 3.4.1 Malaria‐endemic countries

Only four children in the study were born in malaria‐endemic countries (Nigeria and Ghana), moving to the United Kingdom between ages 2 and 5 years old. The remaining 96 children were born primarily in the United Kingdom, as well as in Ireland, the Netherlands, and France. An additional three children had moved to malaria‐endemic countries (Nigeria and Ghana) while <5 years of age, all staying for periods of >1 year before returning to London. Mean spleen length in the seven children who were either born or had lived in malaria‐endemic areas was not significantly higher than the mean spleen length in children born in countries with no malaria (malaria: 9.7 ± 5.3 cm; no malaria: 8.4 ± 3.6 cm, *p* = .4).

#### 3.4.2 Infections with Epstein–Barr virus, cytomegalovirus, and parvovirus B19

Of 91 children with available serology, 57 tested IgG‐positive for EBV. EBV IgG‐positive children were older than IgG‐negative children (IgG‐positive: 9.9 ± 3.9 years; IgG‐negative: 5.2 ± 4.5 years, *p* < .0001). Spleen length in the EBV IgG‐positive group was similar to the IgG‐negative group (IgG‐positive: 8.5 ± 3.8 cm; IgG‐negative: 7.9 ± 3.0 cm, *p* = .4). Adjusting for age in multiple linear regression did not affect this result (*p* = .4). Of 95 children with available serology, 51 tested IgG‐positive for CMV. CMV IgG‐positive children were older than IgG‐negative children (IgG positive: 10.0 ± 4.3 years; IgG negative: 6.5 ± 4.4 years, *p* = .0002). Spleen length in the CMV IgG‐positive group was similar to the IgG‐negative group (IgG‐positive: 8.7 ± 4.4; IgG‐negative: 8.1 ± 2.8 cm, *p* = .4). Adjusting for age in multiple linear regression did not affect this result (*p* = .6). Of 85 children with available serology, 15 tested IgG‐positive for parvovirus B19. Parvovirus B19 IgG‐positive children were older than IgG‐negative children (IgG‐positive: 11.9 ± 3.0 years; IgG‐negative: 7.5 ± 4.6 years, *p* = .0008). Spleen length in the parvovirus B19 IgG‐positive group was similar to the IgG‐negative group (IgG‐positive: 9.3 ± 4.5 cm; IgG‐negative: 8.3 ± 3.7 cm, *p* = .4). Adjusting for age in multiple linear regression did not affect this result (*p* = .6).

#### 3.4.3 Other factors

HbF measurements were available for 95 children. There was no significant correlation between spleen length and %HbF (*r* = .07, *p* = .5). Results were similar when adjusting for age in multiple linear regression (*p* = .3). Spleen length was greater in children with homozygous α‐thalassemia (two α‐globin gene deletions) (10.2 ± 4.4 cm), compared to children with heterozygous α‐thalassemia (one α‐globin gene deletion) (8.7 ± 4.1 cm) or no α‐globin gene deletions (7.9 ± 3.3 cm). This difference did not reach statistical significance (*p* = .3). Data on G6PD enzyme activity were available for 94 children, 10 of whom were deficient. Mean spleen length was similar in children with G6PD‐deficiency and children with normal enzyme activity (deficiency: 9.3 ± 3.9 cm; normal activity: 8.2 ± 3.8 cm, *p* = .4). Results were similar when adjusting for gender in multiple linear regression (*p* = .5). Of the 100 children, 47 were currently receiving HU‐treatment. There was no difference in spleen length in the HU group compared to the non‐HU group (HU: 8.7 ± 3.8 cm; non‐HU: 8.2 ± 3.7 cm, *p* = .5). In the HU group, age at treatment initiation was not significantly associated with spleen length (*p* = .05).

### 3.5 RBC deformability

**FIGURE 2:**
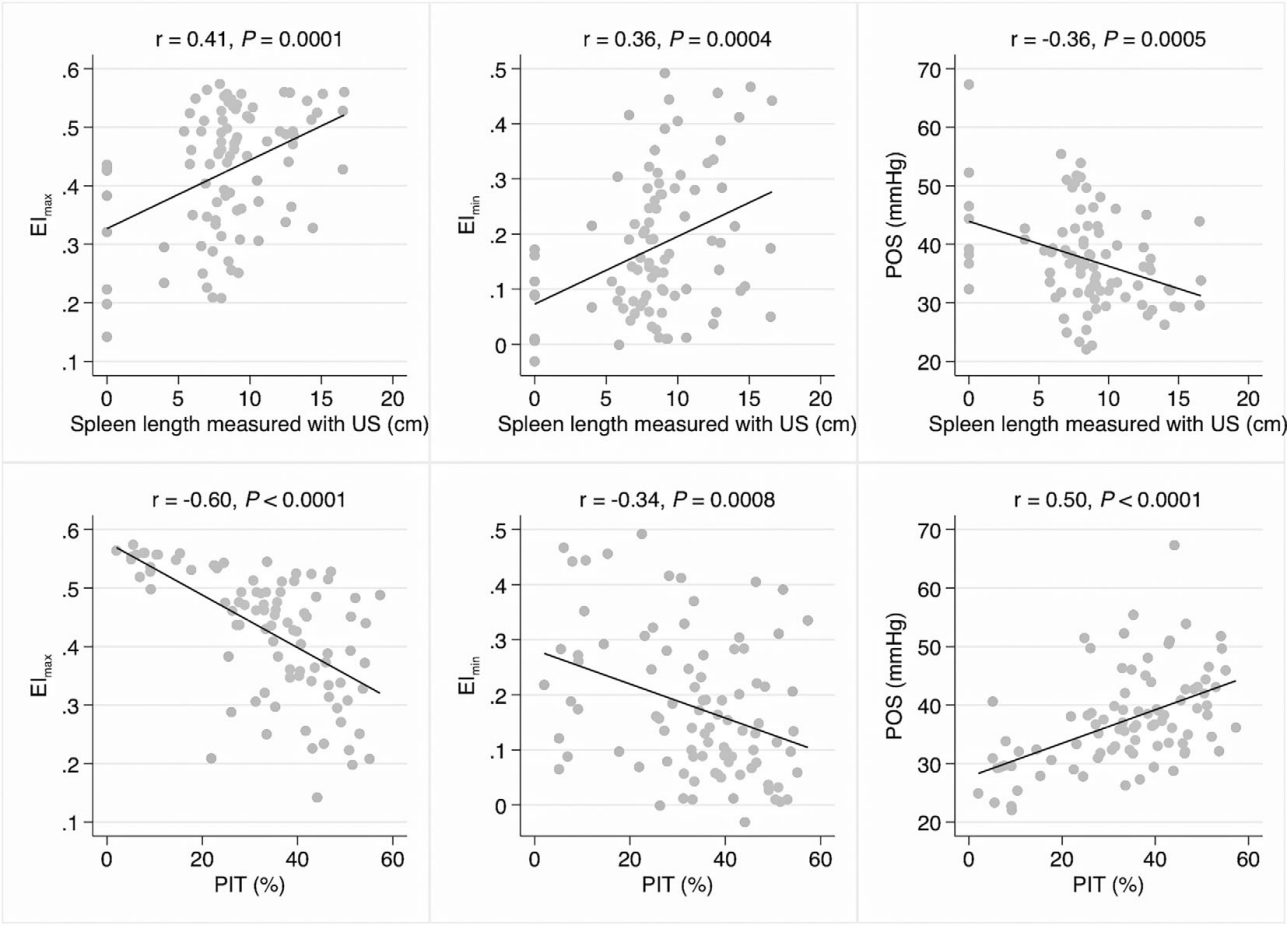
Association between spleen size/splenic function and red blood cell deformability. Top row: Correlation between spleen length, measured with abdominal ultrasound (US), and oxygen gradient ektacytometry‐derived biomarkers. Bottom row: Correlation between splenic function, assessed with pitted red blood cell counts, and oxygen gradient ektacytometry‐derived biomarkers.

Results of oxygen gradient ektacytometry were available for 91 children. Results have previously been presented in detail,^15^ although not in relation to splenic outcomes. We found RBC deformability expressed as the elongation index (EI) at normal oxygen tensions (EI_max_) and after deoxygenation (EI_min_) to correlate positively with spleen length (EI_max_: *r* = .41, *p* = .0001; EI_min_: *r* = .36, *p* = .0004). The point of sickling (POS) correlated negatively with spleen length (*r* = −.36, *p* = .0005). Correlations between spleen length and oxygen gradient ektacytometry results are presented in Figure 2.

Additionally, we found EI_max_ and EI_min_ to both correlate negatively with %PIT (EI_max_: *r* = −.60, *p* < .0001; EI_min_: *r* = −.34, *p* = .0008) and POS to correlate positively with %PIT (*r* = .50, *p* < .0001). Correlations between %PIT and oxygen gradient ektacytometry results are presented in Figure 2. EI_max_, EI_min_, and POS remained significant predictors for %PIT when adjusting for age and %HbF in multiple linear regression models (EI_max_: *p* < .001; EI_min_: *p* = .03; POS: *p* < .001).

### 3.6 Spleen size and clinical outcomes

#### 3.6.1 Acute splenic sequestration

Only five children included in the study had a history of ASS, ranging from 1 to 5 episodes. Four of these children had a spleen length above the suggested upper limit for their age and one had average spleen length for age. We found spleen length to be significantly higher in children with previous ASS (12.5 ± 4.0 cm) compared to children with no history of ASS (8.2 ± 3.6 cm, *p* = .01). Data on spleen volume were available for the five children with previous ASS and 66 with no previous history of ASS. Spleen volume was higher in children with previous ASS (median: 99.6 ml, IQR: 411.2) compared to children with no previous ASS (median: 63.4 ml, IQR: 81), although this result did not reach statistical significance (*p* = .1).

#### 3.6.2 Acute chest syndrome

Of the 100 children included in the study, 22 had a history of ACS. Seven of these children had no visible spleen on US. We found that spleen length was significantly lower in children with a history of ACS (6.6 ± 5.3 cm) compared to children with no previous ACS (9.0 ± 3.0 cm, *p* = .007). Data on spleen volume were available for 17 children with previous ACS and 54 with no previous history of ACS. Spleen volume was lower in children with previous ACS (median: 53.7 ml, IQR: 114.5) compared to children with no previous ACS (median: 66.4 ml, IQR: 93.8), although this result did not reach statistical significance (*p* = .1).

#### 3.6.3 Painful crises

A total of 53 children had experienced one or more episodes of painful crises within 24 months of study inclusion. There was no significant association between history of painful crises and spleen length (+pain: 8.2 ± 4.0 cm; −pain: 8.8 ± 3.5 cm, *p* = .4).

#### 3.6.4 Transcranial Doppler velocities

TCD velocities were available for 91 children. When examining the association between spleen length and the highest recorded TCD velocity for each child, there was no significant correlation (*r* = −.1, *p* = .2).

## 4 DISCUSSION

We conducted a cross‐sectional study examining spleen size and function in children with SCA living in the United Kingdom. We found no correlation between age and spleen size measured with abdominal US, with splenomegaly being common in all age‐groups. In keeping with previous findings,^18^ we found no association between spleen size and splenic function assessed with PIT counts. Despite most children having average spleen sizes for their age, only one child (aged 1 year) had a %PIT <3.5% suggestive of normal splenic function.^2^

In 1935, Diggs first reported on spleen pathology in SCA, describing a series of changes, ranging from splenomegaly to complete fibrotic atrophy (autosplenectomy).^9^ He found no direct correlation between age and spleen size. Although splenomegaly was more frequent in very young children and atrophic spleens in older children and adults, large spleens were sometimes found in adults and small spleens in young children. In 1956, Watson et al. published data on splenomegaly in 115 individuals with SCA.^8^ They found that 35.1% of children <10 years had palpable splenomegaly on clinical examination compared to 10.3% of children ≥10 years and adults. These studies formed the basis of our understanding of the natural history of the spleen in SCA and it has since been the general perception that splenomegaly primarily occurs in young children, with the vast majority undergoing autosplenectomy within the first decade of life.

In a pivotal study from 1969, Pearson et al. described how children with SCA had palpable spleens with no scintigraphic uptake, so‐called “functional asplenia”.^23^ It is now well‐established that splenic function is lost very early in life in children with SCA, with evidence of impaired function in children as young as 6 months.^2^, ^3^, ^24^ Because of this, penicillin prophylaxis and pneumococcal immunizations were introduced for children with SCA living in high‐income settings, dramatically reducing infection rates and improving survival.^25^ Nonetheless, invasive infections with encapsulated bacteria are still the main cause of early childhood mortality in low‐income countries.^26^ It is important to separate the concept of “functional asplenia” from the term “autosplenectomy”. These expressions are often used interchangeably and, although this makes no difference for treatment purposes, it causes confusion as to how many children with SCA have no splenic function and how many children have no detectable spleen.

In the current study, we found that just 5.6% of children aged 6–10 years and 19.4% of children aged 11–16 years had no visible spleen on abdominal US (autosplenectomy). These numbers are lower than we had anticipated, showing that autosplenectomy does not occur within the first decade of life in the majority of children living in our non‐malaria endemic setting. We found splenomegaly to be common at all ages, with similar rates in younger (0–5 years) and older children (11–16 years), accounting for 32.1% and 36.2% in their age groups. When comparing our results to those from other studies examining spleen size with US in children and young adults with SCA living in North America and Europe, findings are similar. Rates of autosplenectomy range from 21% to 42.9% and persistent splenomegaly from 15.1% to 30.1%, with splenomegaly being present in younger children, as well as older children and young adults.^27^, ^28^, ^29^ Persistent splenomegaly has often been associated with milder disease phenotypes,^30^ and it is therefore tempting to conclude that these findings suggest improved outcomes in SCA. However, solid data supporting very high rates of autosplenectomy are sparse, and it is unclear whether spleen size in SCA has changed. Our findings are not so different from those described by Diggs almost 90 years ago and perhaps only reflect how the natural history of splenic injury in SCA is still not fully understood.

Spleens vary enormously in shape. Therefore, spleen volume might be a more meaningful way of assessing spleen size. Measuring spleen volume is slightly more time‐consuming and technically difficult than measuring spleen length, particularly in young children. Furthermore, spleen size measured by US has historically been assessed primarily using spleen length and pediatric normal values are mainly presented as spleen length for age or spleen length for height.^22^, ^31^ We found a very strong correlation between spleen volume and spleen length, a result similar to previous findings,^28^, ^32^ suggesting that US measurements of only spleen length could be sufficient when assessing spleen size in children with SCA. We therefore chose to carry out the majority of analyses in this study using spleen length measurements.

Historically, palpable splenomegaly in older children with SCA has been reported more frequently in studies from SSA, and it has therefore often been attributed to malaria exposure. However, reports on the association between spleen size and malaria infections or parasitemia have come to differing conclusions^11^, ^12^, ^33^, ^34^ and the relationship has not been fully established. Most data reporting on spleen size in SSA are based on abdominal palpation and in studies examining spleen size by US, results are somewhat varied. We found that five separate Nigerian studies in children with SCA aged 6 months to 15 years, reported rates of autosplenectomy between 4.2% and 20% and rates of splenomegaly between 21.1% and 56.5% when measured by US.^33^, ^35^, ^36^, ^37^, ^38^ Most studies described splenomegaly occurring in all age groups and no cases of autosplenectomy in children <8 years. These results are very similar to those in our study, suggesting that perhaps spleen size does not vary significantly between malaria‐endemic and non‐malaria‐endemic settings. In our study, four children were born in malaria‐endemic countries and an additional three children had lived in malaria‐endemic countries for >1 year while under 5 years old. These children did not have significantly larger spleens compared to the remaining cohort. Admittedly, this analysis may be underpowered due to the small sample size. Further examining the effects of malaria on spleen size in SSA will be valuable.

It has been suggested that high levels of HbF are associated with persistent splenomegaly. A study from Saudi Arabia found %HbF to be higher in individuals with marked splenomegaly measured by US compared to individuals with autosplenectomy (22.2% compared to 14.6%).^13^ In contrast, our results found no correlation between spleen length and %HbF. Another factor thought to protect the spleen is co‐inheritance of α‐thalassemia. In a Jamaican population of children and adults with SCA, palpable splenomegaly was reported in 45% of individuals with homozygous α‐thalassemia, 24% with heterozygous α‐thalassemia and only 14% with no α‐globin gene deletions. Although we saw a trend toward increased spleen length in children with homozygous α‐thalassemia, this did not reach statistical significance, potentially due to a lack of power. However, data from Jamaica were based on palpation only, and 14 children in our study had large spleens on US but no palpable splenomegaly. Furthermore, a Nigerian study also examining splenomegaly by abdominal palpation found that co‐inherited α‐thalassemia did not influence spleen size.^34^ Mean spleen length was similar in children with G6PD‐deficiency and children with normal G6PD enzyme activity. Some mild cases of G6PD deficiency, particularly those caused by the A‐variant, might have been missed by the enzyme assay, although these mild cases are less likely to have a marked effect on spleen size and function. Other factors known to potentially affect spleen size are viral infections, such as CMV and EBV. Both CMV and EBV are associated with splenomegaly, albeit usually mild and in relation to acute/subacute infection.^39^, ^40^ To the best of our knowledge, these infections have not previously been examined in relation to spleen size in SCA. We did not find any association between CMV or EBV IgG‐positivity, indicating previous infections with these viruses, and spleen size.

We assessed RBC deformability in 91 children using oxygen gradient ektacytometry and found a strong association with spleen length. Children with larger spleens had improved deformability expressed as higher EI_max_ and EI_min_, as well as a lower POS, suggesting increased RBC tolerance to deoxygenation. We found that oxygen gradient ektacytometry results also correlated with %PIT. This is in agreement with a recent study from France, in which ISCs were found to correlate positively with Howell‐Jolly body counts quantified by flow cytometry, as well as splenic uptake on ^99m^Tc heated RBC spleen scintigraphy.^4^ Although it is difficult to interpret the exact causal relationship between RBC deformability and splenic injury, results indicate that impaired deformability may play a key role in the loss of splenic filtration.

Several previous studies have evaluated the effect of HU treatment on splenic filtration function and results are conflicting.^41^, ^42^, ^43^ Although HU does not have a marked effect on RBC deformability (EI_max_ and EI_min_),^15^ reduced rates of HbS polymerization are likely to have many downstream effects which may improve splenic outcomes. The effect on spleen size, however, is harder to predict. In our study, spleen size did not differ between children treated with or without HU. When we started study recruitment, guidelines in the United Kingdom recommended HU treatment only for children with frequent painful episodes or other SCA‐related complications. Hence, children presenting with more severe phenotypes were more frequently receiving HU than children presenting with milder disease. This may have influenced results. To determine long‐term effects of HU on both spleen size and splenic function, larger prospective studies are necessary.

The clinical consequences of spleen size have not previously been studied in detail and it is not clear whether children with splenomegaly or autosplenectomy are at particular risk of certain complications. In a French study, 79% of children who experienced ASS had no previous history of palpable splenomegaly on clinical examination.^7^ Data were collected retrospectively and, thus, spleen size had not been investigated consistently throughout the cohort. In a North American study on the effects of chronic transfusion, 11 children with a history of ASS and visible spleens on US had significantly smaller spleen volume compared to 103 children with no history of ASS.^28^ In contrast, we found spleen length to be significantly higher in children with a history of ASS. In addition, seven of nine children with autosplenectomy had a history of one or more episodes of ACS and spleen length in children with a history of previous ACS was significantly smaller compared to children with no previous ACS. For both ASS and ACS, spleen volume was not significantly different between the groups, which may be explained by lower power in the volume analysis.

Finally, an inherent limitation to our study is its cross‐sectional design. Although it provides data on the prevalence of autosplenectomy and splenomegaly, it is difficult to infer the factors that are causally related to spleen size without longitudinal data. Some prospective studies have studied splenic function in infants with SCA, but longitudinal data on spleen size are lacking. Further studies comparing spleen size and function in children and adults with SCA in high‐ and lower‐income countries are necessary in order to better understand the natural history of spleen injury and to further explore the impact of genetic and external factors.

## 5 CONCLUSION

In our cohort of children with SCA living in the United Kingdom, we found that autosplenectomy was fairly uncommon before the age of 16, affecting about 20%. Splenomegaly, however, was common in younger as well as older children. These results do not agree with the general perception that most children with SCA undergo autosplenectomy within the first decade of life. Our results are very similar to those from studies in SSA, suggesting that perhaps spleen size does not vary between malaria‐endemic and non‐malaria‐endemic settings, as has previously been hypothesized. Furthermore, our results indicate that loss of erythrocyte deformability contributes to loss of splenic filtration capacity in SCA, as well as phenotypical variations in spleen size. To better understand the natural history of the spleen in SCA, further studies examining the spleen in children and adults living in both high‐ and lower‐income countries are necessary.

## AUTHOR CONTRIBUTIONS

Amina Nardo‐Marino, Andreas Glenthøj, Jørgen A. L. Kurtzhals, Thomas N. Williams, and David C. Rees designed and planned the research study. Amina Nardo‐Marino and John N. Brewin recruited study participants and collected samples. Amina Nardo‐Marino performed all ultrasound examinations. Amina Nardo‐Marino and Thomas H. Braunstein performed all microscopy. Amina Nardo‐Marino analyzed the data and wrote the manuscript. All authors contributed to the final approved version of this report.

## CONFLICT OF INTEREST

Andreas Glenthøj: Consultancy (Agios, Bristol Myers Squibb, Novo Nordisk, and Novartis) and research funding (Sanofi, Saniona, and Alexion).

## Data Availability

The data that support the findings of this study are available from the corresponding author upon reasonable request.
